# Coherent THz Emission Enhanced by Coherent Synchrotron Radiation Wakefield

**DOI:** 10.1038/s41598-018-30125-1

**Published:** 2018-08-03

**Authors:** S. Di Mitri, A. Perucchi, N. Adhlakha, P. Di Pietro, S. Nicastro, E. Roussel, S. Spampinati, M. Veronese, E. Allaria, L. Badano, I. Cudin, G. De Ninno, B. Diviacco, G. Gaio, D. Gauthier, L. Giannessi, S. Lupi, G. Penco, F. Piccirilli, P. Rebernik, C. Spezzani, M. Trovò

**Affiliations:** 1 Elettra–Sincrotrone Trieste S.C.p.A, 34149 Basovizza, Trieste, Italy; 2 Department of Physics, University of Trieste, 34100 Trieste, Italy; 3 University Lille, CNRS, UMR 8523 - PhLAM - Physique des Lasers Atomes et Molécules, 59000 Lille, France; 4 LIDYL, CEA, CNRS, Université Paris-Saclay, Saclay, 91191 Gif-sur-Yvette France; 5 ENEA Frascati, Via E. Fermi 45, 00044 Frascati, Rome Italy; 6 University of Rome – La Sapienza, 00185 P.le A. Moro 2, Rome, Italy; 7 CNR-IOM, 34149 Basovizza, Trieste, Italy

## Abstract

We demonstrate that emission of coherent transition radiation by a ∼1 GeV energy-electron beam passing through an Al foil is enhanced in intensity and extended in frequency spectral range, by the energy correlation established along the beam by coherent synchrotron radiation wakefield, in the presence of a proper electron optics in the beam delivery system. Analytical and numerical models, based on experimental electron beam parameters collected at the FERMI free electron laser (FEL), predict transition radiation with two intensity peaks at ∼0.3 THz and ∼1.5 THz, and extending up to 8.5 THz with intensity above 20 dB w.r.t. the main peak. Up to 80-µJ pulse energy integrated over the full bandwidth is expected at the source, and in agreement with experimental pulse energy measurements. By virtue of its implementation in an FEL beam dump line, this work promises dissemination of user-oriented multi-THz beamlines parasitic and self-synchronized to EUV and x-ray FELs.

## Introduction

THz spectroscopy has been widely employed in the last two decades for research in solid-state-physics, biology, medicine and industrial production. A new frontier is represented by the possibility of generating sub-picosecond coherent THz pulses suitable to manipulate and control material properties. To this aim, pulse energy from µJ to mJ and peak electric field beyond 100 kV/cm are needed. The key advantage of using THz radiation instead of, e.g., visible, is that the former can couple resonantly to electronic, magnetic and vibrational degrees of freedom of compounds, thus offering an extremely wide range of excitations to be explored^1–10^.

In the last decade, THz coherent synchrotron radiation (CSR) from dipole magnets has been collected at last generation storage ring light sources^11–20^. In spite of peak electric field well below the kV/cm-level and of the relatively low peak power, a high average photon power is obtained by virtue of the ∼1–500 MHz pulse repetition rate. Production of pulses as short as ∼1 picosecond requires a dedicated electron optics for the storage of few, high charge bunches. This optics, however, is not deemed to be compatible with the standard multi-bunch filling pattern of those user facilities. Sub-picosecond photon pulses can be produced by means of the so-called femto-slicing technique^21–25^. The short pulse duration is obtained at the expense of at least 4 orders of magnitude lower peak photon flux w.r.t. standard synchrotron radiation emission, and repetition rates typically lower than ∼100 kHz.

In the last few years significant advances in laser-based THz techniques, namely optical rectification and optical parametric amplifiers, have demonstrated the possibility to achieve the pulse energies required for nonlinear studies over the full THz range^26–28^. Unfortunately, organic crystals used as THz emitters, especially in the 1–10 THz range, suffer from limitations in terms of peak power and repetition rate due to their low radiation damage threshold; they are also limited in bandwidth due to the presence of phonon resonances responsible for the THz gaps. Generation of THz in air and noble gases using a laser-gas interaction^29–31^ might overcome the bandwidth limit and the damage threshold, at the expense of photon pulse energies typically below the μJ-level.

A THz source based on a radiofrequency linear accelerator (linac)^32–39^ does not suffer, in principle, from any of the above-mentioned limitations in terms of peak electric field, pulse energy and duration. In the last decade, electron linacs have been built in order to shorten the electron bunch to sub-millimeter lengths, so emitting THz radiation in the so-called super-radiant regime^40^. A dedicated linac-based THz source can provide radiation with high average power (e.g., by virtue of superconducting RF linac technology) and narrow bandwidth (e.g., when THz emission is generated in an undulator). Unfortunately, construction of such a dedicated THz source implies costs commonly much larger than those of a laser-based one.

This study demonstrates that coherent transition radiation (CTR) with state-of-the-art features – sub-picosecond duration, up to nearly 80-µJ pulse energy in the THz gap – was produced by exploiting the electrons’ interaction with CSR emitted in the post-undulator dispersive region of the EUV FERMI free electron laser (FEL)^41,42^. THz radiation with those characteristics finds application in experiments involving, for example, resonantly exciting collective modes as phonons, polarons, charge/spin-density-wave gaps, superconducting gaps in high-temperature superconductors and plasmons.

**Figure 1:**
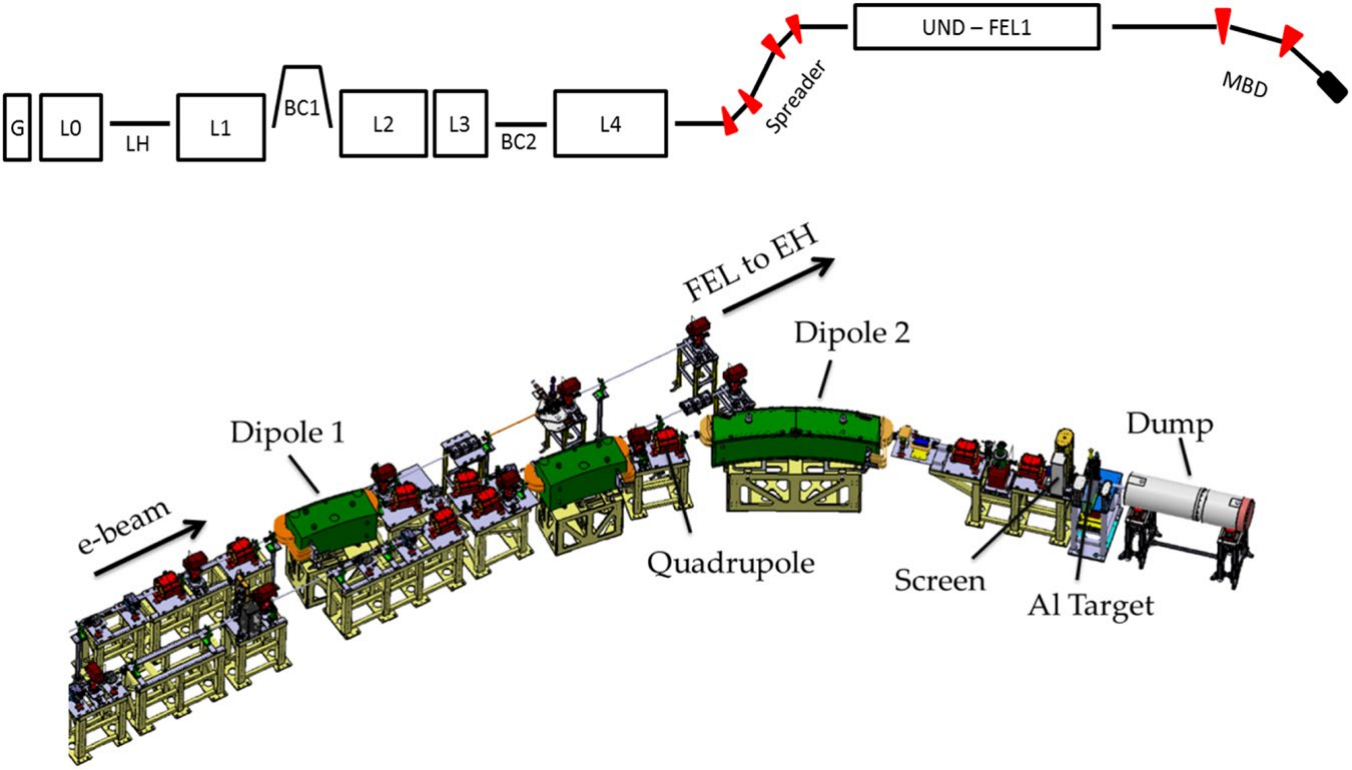
Top: sketch, not to scale, of the FERMI FEL1 beam delivery system. Only the first magnetic bunch length compressor (BC1) is routinely active for lasing. Start-to-end simulations of the electron beam dynamics were carried out, for diverse linac settings, from the Gun (G) through the linac sections (L0–L4) until the Main Beam Dump (MBD), where the Al target for CTR emission is installed. Bottom: 3-D rendering of the MBD, from the FEL post-undulator region to the dump. The active dipole magnets, named “Dipole 1” and “Dipole 2” in the figure, are long 1.12 m and 2.44 m, with bending angles of 15.7 deg and 31.4 deg respectively. Three quadrupoles between “Dipole 1” and “Dipole 2” tune the momentum compaction (R_56_) of the beam line. CTR is emitted at the 1 µm-thick Al target. Four quadrupoles installed upstream of the first dipole control the beam envelope along the line without affecting the R_56_ value. The transverse RMS beam sizes at the CTR target are kept smaller than 0.5 mm. Steering magnets and beam position monitors (not shown) allow control of beam trajectory. While electrons are bent and eventually dumped, FEL propagates straight to the downstream experimental hall (EH).

A sketch of the FERMI electron beam delivery system is shown in Fig. 1. Since the electron beam manipulation for CTR emission occurs after lasing, the scheme allows the TeraFERMI photon beamline^43,44^ to run in parasitic mode to the FEL. The THz pulse is self-synchronized to the FEL emission thus opening, in principle, a wide range of scientific applications in pump-probe configurations. By virtue of the relatively simple set up, the scheme promises dissemination of user-oriented multi-THz beamlines at existing and planned FEL infrastructures.

## Concept

CTR is generated by a ∼1 GeV energy-electron beam passing through a 1 µm-thick Al foil, at the repetition rate of 10 or 50 Hz. The foil is installed at the end of the post-undulator Main Beam Dump line (MBD) shown in Fig. 1. Backward-emitted CTR is collected in vacuum by the TeraFERMI photon transport line^45^. The full width duration of the electron bunch prepared for lasing, thus entering the MBD line, is approximately 1 picosecond full width. In order to maximize transition radiation intensity at frequencies of 1 THz and above, a sub-picosecond charge density structure, i.e., a kA-level current spike is generated in the bunch passing through the MBD, before reaching the Al target. Doing so, radiation is emitted coherently in the multi-THz range at several tens of µJ-energy per pulse, as shown below.

Three quadrupole magnets allow the tuning of the MBD linear momentum compaction (R_56_)^46^. This determines the electron bunch length compression factor (CF) that, at first order in the particle coordinates, results:

1 $$CF(z)=1/|1+{h}_{1}{R}_{56}|$$

**Figure 2:**
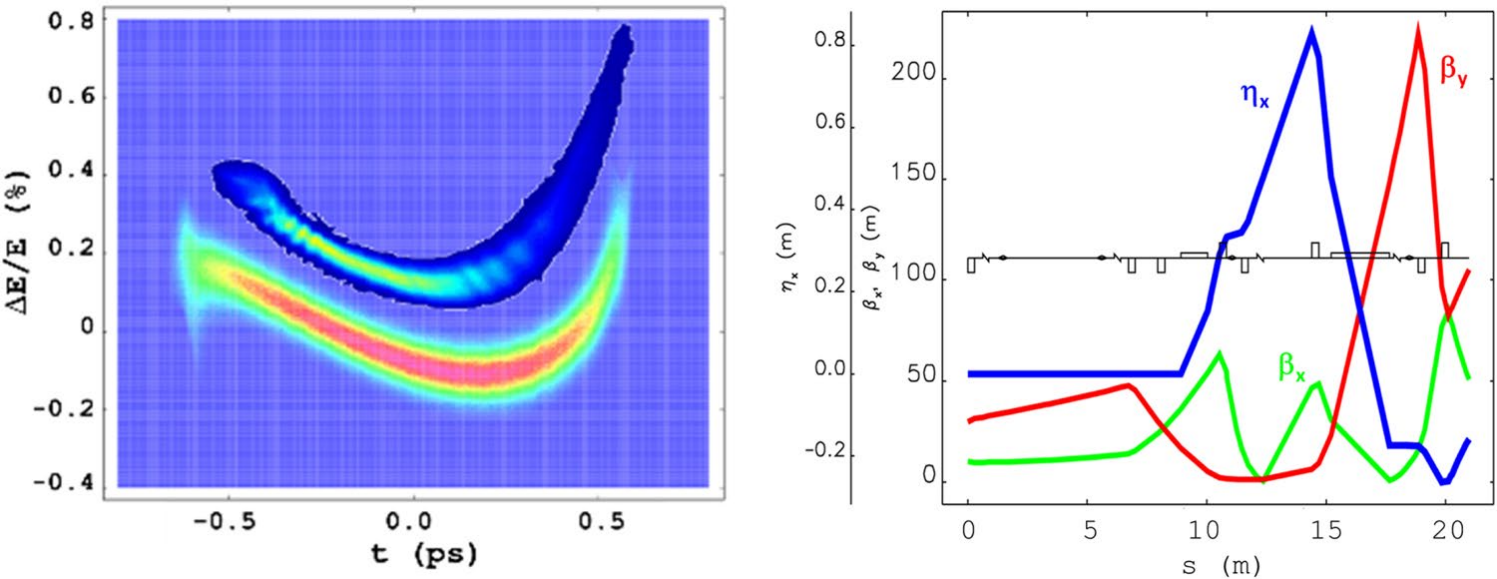
Left: simulated and measured electron beam longitudinal phase space at the end of the FERMI linac, at the beam energy of 1308 MeV (see also Table 1); bunch head is at negative time coordinates. The comparison reveals very similar mean energy, energy spread, pulse duration, linear and higher order energy chirp components. The current profile is approximately flat at the level of ∼600 A (the measured phase space is upper-shifted w.r.t. the simulation for visualization). Right: betatron functions (β_x,y_) and horizontal energy-dispersion function (η_x_) from the exit of the FERMI undulator to the Al target (see Fig. 1). These functions were calculated starting from Twiss parameters measured in front of the undulator, and for the actual quadrupoles setting. They correspond to R_56_ = 19 mm. Measured (simulated) RMS beam sizes at the screen in front of the target were: 0.37 (0.33) ± 0.03 mm horizontal, 0.09 (0.08) ± 0.03 mm vertical.

${h}_{1}={E}_{b}^{-1}(dE/dz)$ is the initial linear correlation of the particle energy, *E*, with the particle longitudinal position internal to the bunch, *z*. This quantity, also named “linear energy chirp”, is normalized to the beam mean energy, ${E}_{b}$. The energy chirp at the entrance of the MBD contains linear and quadratic terms induced by linac wakefields^47,48^ (see later Fig. 2). The chirp, however, further evolves along the line because of the interaction of leading electrons with CSR emitted by trailing ones, as the bunch propagates through dipole magnets. The longitudinal electric field associated to such a CSR tail-head interaction is often referred to as CSR wakefield. The energy chirp is eventually dominated by this interaction.

In its steady-state formulation^49,50^, the CSR-induced beam RMS relative energy spread is

2 $${\sigma }_{\delta ,CSR}\propto Q\theta {R}^{\frac{1}{3}}/(\gamma {\sigma }_{z}^{4/3})$$

with R the dipole magnet bending radius, θ the bending angle, *γ* the beam energy Lorentz factor, *Q* the electron bunch charge involved in coherent emission, and *σ*_*z*_ the RMS bunch length. The single particle energy deviation is actually correlated with *z* (from which one derives the dependence of energy spread from bunch length shown above) and, in particular, a negative linear chirp ${h}_{1} < 0$ is produced at the bunch head, i.e., leading particles are moved to higher energy with respect to trailing ones. Inspired by the idea of using the CSR wakefield to manipulate the electron beam energy distribution^51^, the FERMI dump line was designed to enhance the CSR wakefield by adopting relatively long and large angle dipole magnets, and for producing a tuneable but yet relatively large and positive R_56_. This term couples with the CSR-induced negative energy chirp (see Eq. 1) and, as shown in the following, generates a ∼100 fs-long leading current spike that radiates coherently in the THz range.

## Theoretical and Experimental Results

In a preparatory phase to lasing, the duration of the electron beam produced by the FERMI injector was time-compressed by a factor 8 in a magnetic chicane (BC1 in Fig. 1) at the beam energy of ∼280 MeV. The electron bunch longitudinal phase space was measured at the linac end (exit of L4 in Fig. 1), at the energy of 1308 MeV, by means of a vertical RF deflector in combination with a horizontal spectrometer magnet^52^. The RF deflector translates the particle vertical coordinate, imaged at a downstream screen, into time coordinate, from which the bunch length information can be recovered after proper calibration of the deflecting strength. The spectrometer magnet excites energy dispersion that allows one to relate the horizontal coordinate at the screen to particle energy. The measured phase space was faithfully reproduced in start-to-end particle tracking runs done with GPT code^53^ (for the linac injector) and elegant code^54^, see left plot of Fig. 2. Tracking was successively carried out until the Al target, including geometric and longitudinal space charge impedance as well as CSR wakefield in dipole magnets^55^. Simulations confirm that the phase space does not change from the linac end to the entrance of the dump line.

At the linac exit, the linear energy chirp in the head of the bunch was approximately −2 MeV/ps. The energy chirp induced by the CSR wakefield in the two dipole magnets shown in Fig. 1, for beam parameters in Table 1, is predicted to be −6 MeV/ps or $\,{h}_{1,CSR}\approx -\,23\,{m}^{-1}$, and therefore it dominates the time-compression of the bunch head through the dump line. An optics setting which provides R_56_ = 20 mm, for instance, is expected to increase the bunch head peak current by a factor ∼2, thus reaching the kA-level at the target.

**Table 1:** Electron beam parameters of the THz experimental sessions. The asterisc (*) means “at the linac end”.

Parameter	Value		Units
Bunch Charge	0.7		nC
Initial Bunch Duration	2.8		ps
Mean Energy*	1308	871	MeV
Hor. Norm. Emittance*	2.1	1.8	µm
Vert. Norm. Emittance*	1.7	1.5	µm
Compression Factor*	8	8, 11	
Bunch Duration*	0.35	0.35, 0.25	ps
Peak Current*	560	560, 750	A
Transverse Sizes at the Al target	<0.5 (x), <0.2 (y)		mm
R_56_ of the dump line	5, 19	37	mm

While control of the beam longitudinal dynamics relies, as said, on the benchmarking of the longitudinal phase space at the linac end, the beam transverse motion was kept under control by measuring the transverse emittances and the optical Twiss parameters in front of the undulator with the quadrupole scan technique^56^. Those parameters were tracked down to the dump line in accordance to the experimental quadrupoles’ setting, and are shown in the right plot of Fig. 2. The simulated transverse beam sizes at a screen installed 0.3 m upstream of the Al target (see Fig. 1) were found in agreement with the experimental ones, as depicted in the caption of Fig. 2. It is worth pointing out that the horizontal beam size at the screen in proximity of the Al target is expected to be dominated by the energy dispersion function, *η*_*x*_, in accordance to the setting of the MBD magnetic lattice, and by the beam energy spread: ${\sigma }_{x}\cong {\eta }_{x}{\sigma }_{\delta }$. The integral of the dispersion function through the dipoles sets the momentum compaction according to ${R}_{56}={\int }_{0}^{L}\frac{{\eta }_{x}(s)}{R(s)}ds$. The beam energy spread is expected to be dominated by the CSR-induced energy chirp. Thus, the good agreement of measured and modelled horizontal beam size at the MBD screen is a confirmation of both correct optics setting of the line, of the modelled CSR interaction, and consequently of the modelled bunch length manipulation. Since the dispersion function is non-zero at the Al target, some contribution from $\,{R}_{51}\,$and ${R}_{52}\,\,$to the final bunch shape is also expected. Tracking runs confirm, however, that their effect is negligible w.r.t the action of ${R}_{56}$.

Table 1 summarizes the electron beam parameters and the accelerator setting discussed so far. Two additional linac configurations at lower beam energy are also shown, and discussed below. For each configuration, the aforementioned control of longitudinal phase space and optics was accomplished.

CTR pulse energy and spectrum were calculated by applying the generalized Ginzburg-Frank formula, corrected by the prescription for far-field emission^57^, to the electron bunch distribution simulated at the Al target. The expected value of the photon pulse energy was compared with the one measured by means of a pyroelectric detector installed after a Diamond window, in proximity of the target.

**Figure 3:**
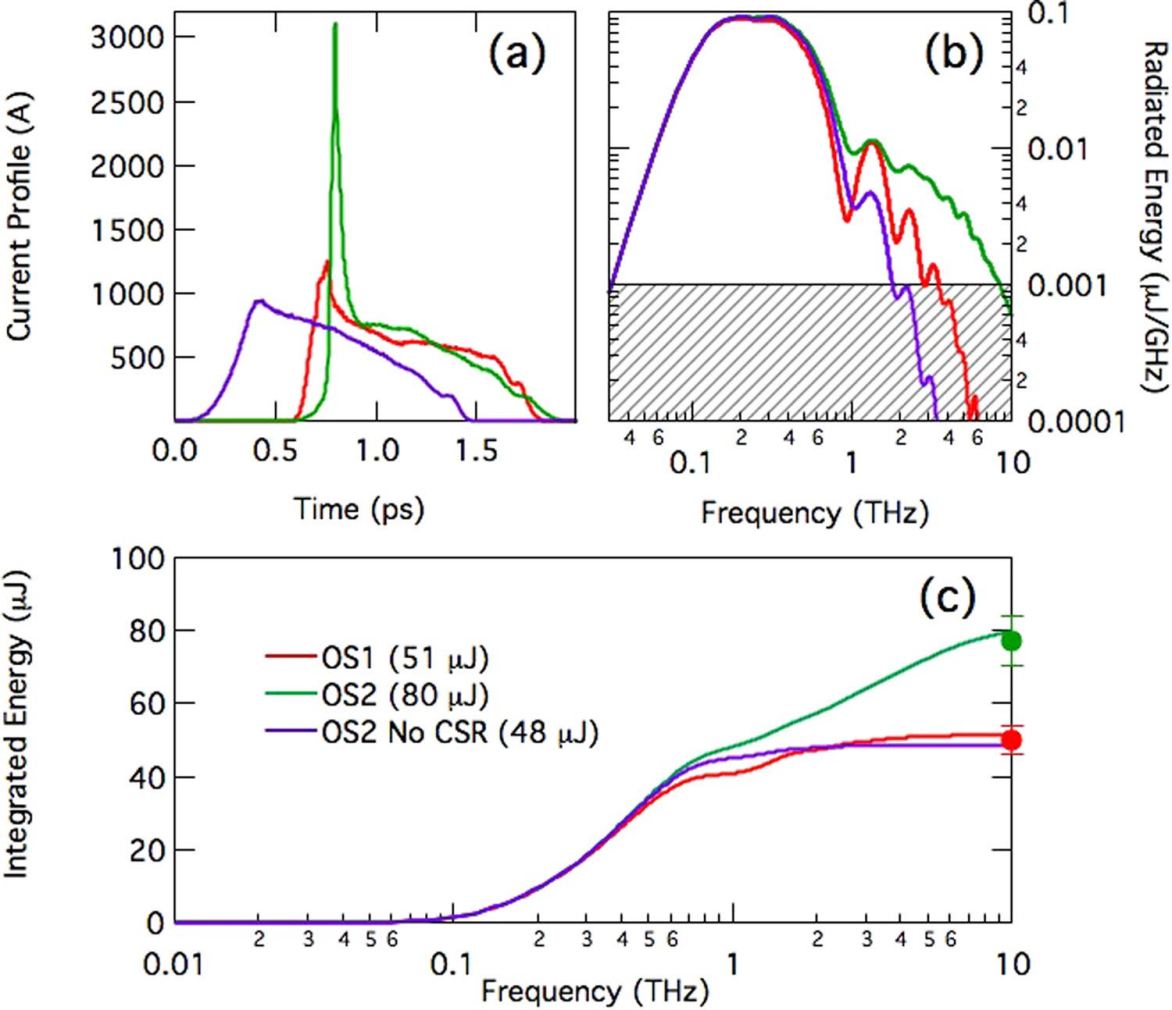
(**a**) Simulated electron bunch current profiles at the Al target for two optics settings of the dump line (OS1-red and OS2-green). For comparison, a case without CSR in the dump line dipole magnets is shown (violet), see also Table 2. (**b**) Calculated CTR spectra. A threshold of 20 dB from the main intensity peak is indicated by the gray area. (**c**) Solid lines: CTR pulse energy integrated over the frequency range 0.01–10 THz and calculated from the above current profiles. Dots with error bars: measured pulse energy, see also Table 2. The pulse energy values refer to emission at the Al foil, i.e., actual measurements are reduced by ∼30% from the shown values because of the transmission efficiency of the Diamond window installed between the foil and the pyroelectric detector. Electron beam and linac parameters are in Table 1, for the beam energy E = 1308 MeV at the linac end.

**Figure 4:**
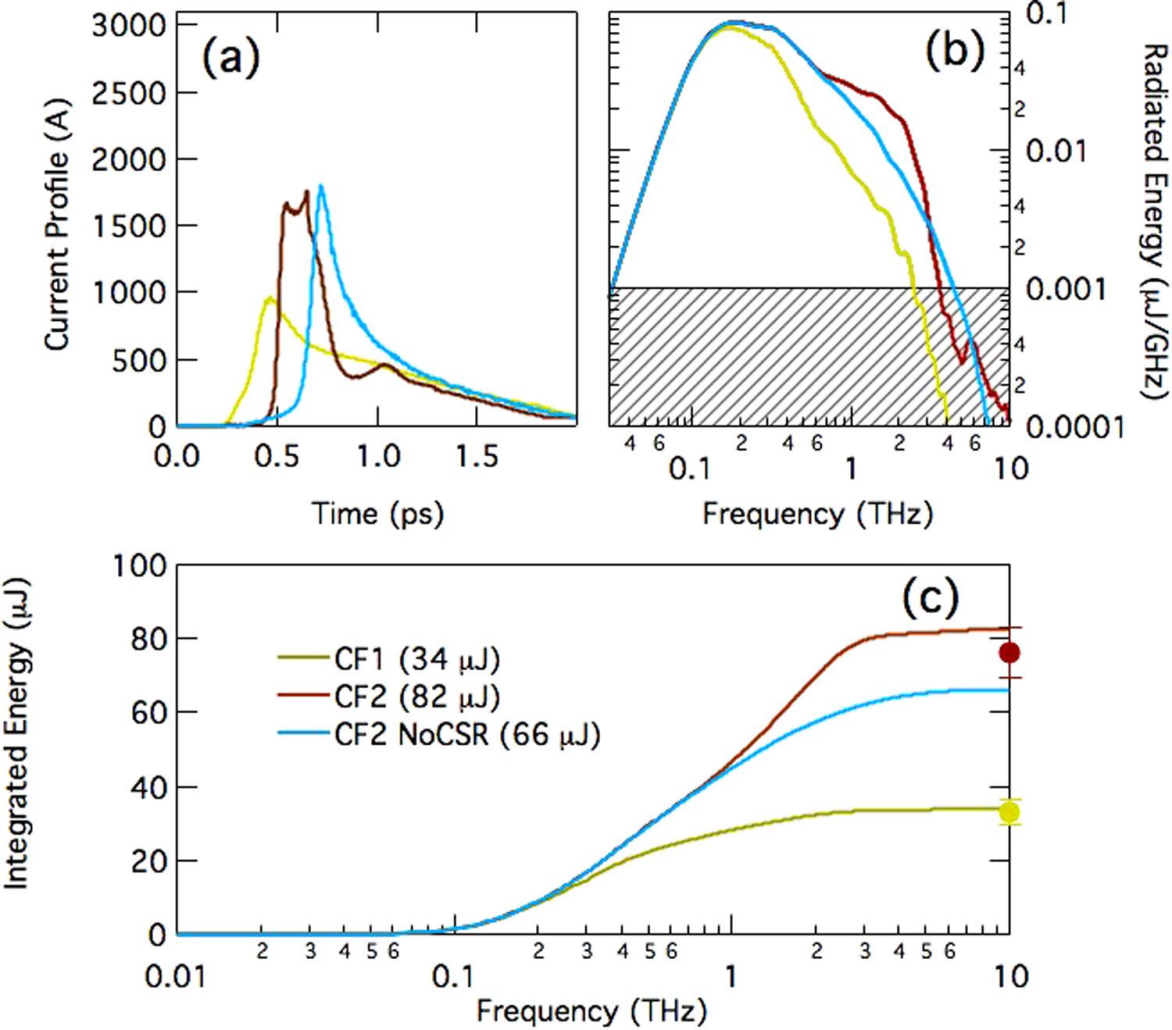
(**a**) Simulated electron bunch current profiles at the Al target for two compression factors in the linac (CF1-yellow and CF2-brown) and fixed R_56_ in the dump line. For comparison, a case without CSR in the dump line dipole magnets is shown (cyan), see also Table 2. (**b**) Calculated CTR spectra. A threshold of 20 dB from the main intensity peak is indicated by the gray area. (**c**) Solid lines: CTR pulse energy integrated over the frequency range 0.01–10 THz and calculated from the above current profiles. Dots with error bars: measured pulse energy, see also Table 2. The pulse energy values refer to emission at the Al foil, i.e., actual measurements are reduced by ∼30% from the shown values because of the transmission efficiency of the Diamond window installed between the foil and the pyroelectric detector. Electron beam and linac parameters are in Table 1, for the beam energy E = 871 MeV at the linac end.

In order to demonstrate that, as predicted by the model, a larger R_56_ increases the bunch head peak current and therefore the THz radiation intensity, pulse energies were measured for two optics settings of the dump line, named “OS1” (R_56_ = 5 mm) and “OS2” (R_56_ = 19 mm), at fixed CF in the linac (beam and linac parameters in Table 1, E = 1308 MeV). Figure 3a shows the electron beam current profiles corresponding to OS1 and OS2, simulated at the target starting from the measurement shown in Fig. 2 -left plot, plus an unphysical case *without* CSR. A 3 kA, 100 fs-long current spike is obtained for OS2. In this case, the CTR calculation in Fig. 3b reveals that a significant frequency content is present up to 8.5 THz. Namely, at 8.5 THz the emission still contains 0.5 μJ energy over a 10% bandwidth, a value which is plausibly high enough to induce nonlinear effects in matter. The theoretical pulse energies integrated over the bandwidth 0.01–10 THz and represented by solid lines in Fig. 3c, are compared with the measured ones (dots with error bar). A striking agreement is achieved only when CSR is included in the simulations. It is worth mentioning that the multi-THz frequency content predicted in Figs 3b and 4b reflects typical spectral measurements collected during TeraFERMI operation, see for example Supplementary Information and Fig. 3 in^44^.

To additional highlight the relevance of the CSR wakefield for the production of THz radiation, another series of measurements was carried out with a fixed R_56_ in the dump line (beam and linac parameters in Table 1, E = 871 MeV), and for two values of the linac CF, named “CF1” (CF = 8) and “CF2” (CF = 11). A shorter bunch at the entrance of the dump line is expected to emit stronger CSR field. This produces a larger negative chirp and therefore a higher leading current spike, thus more intense THz emission. Figure 4a shows two current profiles simulated at the Al target for the two values of the linac CF, plus an unphysical case *without* CSR. Similarly to Fig. 3c, the measured pulse energies in Fig. 4c confirm the expectations only when CSR is included in the simulations.

**Figure 5:**
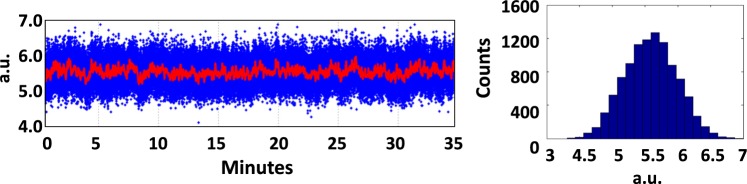
Left: TeraFERMI pyroelectric detector signal (red line is the moving average signal) vs. time. Right: statistical distribution of counts. The RMS fluctuation over 35 minutes is 7%. Typical fluctuations during FEL operation are smaller than 10%.

A summary of the experimental settings of the dump line, of the simulated and of the measured THz-pulse energy is given in Table 2. The pulse energy values refer to emission at the Al foil, i.e., actual measurements are reduced by ∼30% from the shown values because of the transmission efficiency of the Diamond window (transmission efficiency is ∼70% at all wavelengths of interest here) installed between the foil and the pyroelectric detector. The uncertainty on the pulse energy value is dominated by the RMS fluctuation of the THz signal collected by the detector, which is typically smaller than 10%. A representative long-term behaviour of the THz signal is shown in Fig. 5. The striking agreement of measured and predicted pulse energies for all the cases considered, and the energy reduction found in the simulation when CSR is not included, are a conclusive demonstration of the validity of our modelling.

**Table 2:** Momentum compaction of the beam dump line (R_56_), bunch duration at its entrance (σ_t,i_), measured and simulated pulse energies in the 0.01–10 THz range. Pulse energies simulated without CSR are in italics. The error on the measured energy value is dominated by the signal RMS fluctuation.

Configuration	R_56_ [mm]	σ_t,i_ [ps]	E [µJ] simul.	E [µJ] meas.
OS1	5	0.35	51	50 ± 4
OS2	19	0.35	80	77 ± 7
*OS2 No CSR*	*19*	*0.35*	*48*	
CF1	37	0.35	34	33 ± 3
CF2	37	0.25	82	76 ± 7
*CF2 No CSR*	*37*	*0.25*	*66*

## Conclusions and Final Remarks

Production of intense and multi-THz sub-picosecond CTR is predicted by exploiting the CSR wakefield in the dump line of the FERMI FEL. Coherent transition radiation with two intensity peaks at ∼0.3 THz and ∼1.5 THz, and extending up to 8.5 THz with intensity above 20 dB w.r.t. the main peak, is expected. Calculations of THz emission, starting from experimental electron beam parameters of the FERMI FEL, predict up to 80-µJ pulse energy integrated in the range 0.01–10 THz.

The enhancement of the photon pulse energy as a function of the momentum compaction of the FERMI dump line and of the incoming bunch length was verified experimentally. The systematic agreement of measured and calculated pulse energy for different accelerator and dump line optics settings confirms the fundamental role played by the CSR wakefield in generating a ∼0.1 ps long, ∼kA level-current spike in the bunch head, when a positive linear momentum compaction is established. We have therefore shown that the CSR wakefield provides a cost-effective and easy-to-implement technique to compress further the electron bunch, or a portion of it, downstream the FEL emission. As a result, this study demonstrated the independent tuning of THz and EUV FEL emission, thus the parasitic mode of operation of TeraFERMI with respect to the FEL.

In a wider perspective, the demonstrated manipulation of the electron beam after FEL emission goes in the direction of enlarging in a practical and cost-effective way the number of simultaneous users at a linac-driven light source^58,59^. A dissemination of coherent THz sources at existing and planned FEL user facilities in the 1–2 GeV beam energy range is envisioned. An extension of the scheme to higher beam energies is possible, as discussed here in brief. The CSR-induced relative energy chirp is proportional to peak current over beam energy. While soft and hard x-ray FELs^60–64^ run at beam energies as high as 2–17 GeV, they also require bunch peak currents commonly in the range 3–10 kA, in order to drive intense FEL emission in the x-rays. The aforementioned scaling implies that a CSR-induced energy chirp comparable to or larger than the one presented in this study (compare with parameters in Table 1) can be produced even at the highest beam energies. Values of R_56_ of the order of several tens of millimeters can be obtained with standard magnet technology. At the same time, some limited control of the bunch length after lasing has to be expected because of chromatic aberrations related to the large energy spread induced by highly efficient FEL emission.

This study paves the way to additional electron beam manipulation in FEL post-undulator region, such as the adoption of a metallic corrugated structure for generating either a profound linear energy chirp^65–67^ or a THz-scale energy modulation^68^, and successive conversion to density modulation through an energy-dispersive magnetic lattice, for quasi-monochromatic, multi-THz CTR emission. This scheme is presently under study for an upgrade of the TeraFERMI performance.

### Data Availability Statement

The datasets generated during and/or analysed during the current study are available from the corresponding author on reasonable request.

## Electronic supplementary material

Supplementary Information

## References

[1] Tudosa I (2004). The ultimate speed of magnetic switching in granular recording media. Nature (London).

[2] Rini M (2007). Control of the electronic phase of a manganite by mode-selective vibrational excitation. Nature (London).

[3] Först M (2011). Driving magnetic order in a manganite by ultrafast lattice excitation. Phys. Rev. B.

[4] Först, M. *et al*. Nonlinear phononics as an ultrafast route to lattice control. *Nat. Phys. B*, **854** (2011).

[5] Dienst A (2011). Bi-directional ultrafast electric-field gating of interlayer charge transport in a cuprate superconductor. Nat. Photon..

[6] Fausti D (2011). Light-induced superconductivity in a stripe-ordered cuprate. Science.

[7] Liu M (2012). Terahertz-field-induced insulator-to-metal transition in vanadium dioxide metamaterial. Nature (London).

[8] Di Pietro P (2013). Observation of Dirac plasmons in a topological insulator. Nat. Nanotech..

[9] Autore M (2015). Plasmon-phonon interactions in topological insulator microrings. Adv. Opt. Mat..

[10] Giorgianni F (2016). Strong nonlinear terahertz response induced by Diurac surface states in Bi_2_Se_3_ topological insulator. Nat. Commun..

[11] Carr GL, Kramer SL, Murphy JB, Lobo RPSM, Tanner DB (2001). Observation of coherent synchrotron radiation from the NSLS VUV ring. Nucl. Instrum. Methods Phys. Res., Sect. A.

[12] Abo-Bakr M, Feikes J, Holldack K, Wüstefeld G, Hübers H-W (2002). Steady-State Far-Infrared Coherent Synchrotron Radiation detected at BESSY 2. Phys. Rev. Lett..

[13] Byrd JM (2002). Observation of Broadband Self-Amplified Spontaneous Coherent Terahertz Synchrotron Radiation in a Storage Ring. Phys. Rev. Lett..

[14] Mochihashi, A., Hosaka, M., Katoh, M., Shimada, M. & Kimura, S. Observation of THz synchrotron radiation burst in UVSOR-II electron storage ring, In Proc. of the 10^th^ European Particle Accelerator Conference, Edinburgh, UK (2006), THPLS042, p. 3380 (2006).

[15] Karantzoulis E, Penco G, Perucchi A, Lupi S (2010). Characterization of coherent THz radiation bursting regime at Elettra. Infrared Phys. Technol..

[16] Mathis Y-L, Gasharova B, Moss D (2003). Terahertz radiation at ANKA, the new synchrotron light source in Karlsruhe. J. Biol. Phys..

[17] Feikes J (2011). Metrology Light Source: The first electron storage ring optimized for generating coherent THz radiation. Phys. Rev. Special Topics – Accel. Beams.

[18] Martin IPS, Rehm G, Thomas C, Bartolini R (2011). Experience with low-alpha lattices at the Diamond Light Source. Phys. Rev. Special Topics – Accel. Beams.

[19] Tordeux, M.-A. *et al*. Low-alpha operation for the SOLEIL storage ring, In Proc. of the 2012 International Part. Accel. Conf. 1608 (2012).

[20] Ungelenk P (2017). Continuously tunable narrowband pulses in the THz gap from laser-modulated electron bunches in a storage ring. Phys. Rev. Accel. Beams.

[21] Holldack K, Khan S, Mitzner R, Quast T (2006). Femtosecond Terahertz Radiation from Femtoslicing at BESSY. Phys. Rev. Lett..

[22] Byrd JM (2006). Tailored Terahertz Pulses from a Laser-Modulated Electron Beam. Phys. Rev. Lett..

[23] Müller F (2012). Electro-optical measurement of sub-ps structures in low charge electron bunches. Phys. Rev. Special Topics – Accel. Beams.

[24] Shimada M (2007). Laser Bunch Slicing at UVSOR-II Electron Storage Ring. Jpn. J. Appl. Phys..

[25] Labat M (2018). Commissioning of a multi‐beamline femtoslicing facility at SOLEIL. J. Synch. Rad..

[26] Brabec T, Krausz F (2000). Intense few-cycle laser fields: Frontiers of nonlinear optics. Rev. Mod. Phys..

[27] Hauri CP, Ruchert C, Vicario C, Ardana F (2011). Strong-field single-cycle THz pulses generated in an organic crystal. Appl. Phys. Lett..

[28] Fülöp JA (2012). Generation of sub-mJ terahertz pulses by optical rectification. Opt. Lett..

[29] Xie X, Dai J, Zhang X-C- (2006). Coherent Control of THz Wave Generation in Ambient Air. Phys. Rev. Letters.

[30] Kim KY, Taylor AJ, Glownia JH, Rodriguez G (2008). Coherent control of terahertz supercontinuum generation in ultrafast laser–gas interactions. Nat. Photonics.

[31] Kuk D (2016). Generation of scalable terahertz radiation from cylindrically focused two-color laser pulses in air. Appl. Phys. Lett..

[32] Oepts D, van der Meer AFG, van Amersfoort PW (1995). The Free-Electron-Laser user facility FELIX. Inf. Phys. Tech..

[33] Ortega JM, Glotin F, Prazeres R (2006). Extension in far-infrared of the CLIO free-electron laser. Inf. Phys. Tech..

[34] Winnerl, S. *et al*. *FELBE Free-Electron Laser: Status and Application for Time Resolved Spectroscopy Experiments*, Joint 31st International Conference on Infrared and Millimeter Waves and 14th International Conference on Terahertz Electronics, Shanghai, China (2006). IEEE Conference Proceedings, **159**, 1-4244-0400-2 (2007).

[35] Gensch M (2008). New infrared undulator beamline at FLASH. Infrared Phys. Technol..

[36] Shen Y (2011). Tunable Few-Cycle and Multicycle Coherent Terahertz Radiation from Relativistic Electrons. Phys. Rev. Letters.

[37] Chiadroni E (2013). The SPARC linear accelerator based terahertz source. Appl. Phys. Lett..

[38] Wu Z (2013). Intense terahertz pulses from SLAC electron beams using coherent transition radiation. Rev. Sci. Instrum..

[39] Green B (2016). High-Field High-Repetition-Rate Sources for the Coherent THz Control of Matter. Sci. Reports.

[40] Gensch, M. *Super-radiant Linac-based THz Sources in* 2013, in Proc. the 35th Intern. Free Electron Laser Conf., 474–476, New York, NY, USA (2013).

[41] Allaria E (2012). Highly coherent and stable pulses from the FERMI seeded free-electron laser in the extreme ultraviolet. Nat. Photon..

[42] Allaria E (2013). Two-stage seeded soft-X-ray free-electron laser. Nat. Photon..

[43] Perucchi A, Di Mitri S, Penco G, Allaria E, Lupi S (2013). The TeraFERMI terahertz source at the seeded FERMI free-electron-laser facility. Rev. Sci. Instrum..

[44] Di Pietro P (2017). TeraFERMI: A Superradiant Beamline for THz Nonlinear Studies at the FERMI Free Electron Laser Facility. Synchr. Rad. News.

[45] Svetina C (2016). Photon transport of the superradiant TeraFERMI THz beamline at the FERMI free-electron laser, J. Sync. Rad.

[46] Brown, L. A First- and Second-Order Matrix Theory for the Design of Beam Transport Systems and Charged Particle Spectrometers, SLAC Report 75 (1982).

[47] Penco G (2014). Experimental Demonstration of Electron Longitudinal-Phase-Space Linearization by Shaping the Photoinjector Laser Pulse. Phys. Rev. Letters.

[48] Shafqat N, Di Mitri S, Nicastro S, Serpico C (2017). Design study of high gradient, low impedance accelerating structures for the FERMI free electron laser linac upgrade. Nucl. Instrum. Meth. Phys. Res. A.

[49] Derbenev, Y. S., Rossbach, J., Saldin, E. L. & Shiltsev, V. D. Microbunch Radiative Tail-Head Interaction, TESLA-FEL 95-05, DESY. Hamburg, Germany (1995).

[50] Saldin EL, Schneidmiller EA, Yurkov MV (1997). On the coherent radiation of an electron bunch moving in an arc of a circle,. Nucl. Instrum. Meth. Phys. Research, Sect. A.

[51] Bosch RA (2010). Reduction of energy chirp by the wake of coherent synchrotron radiation. Phys. Rev. Special Topics – Accel. Beams.

[52] Craievich P (2015). Implementation of Radio-Frequency Deflecting Devices for Comprehensive High-Energy Electron Beam Diagnosis, IEEE Trans. Nucl. Science.

[53] vanderGeer, S. B. *et al*. http://www.pulsar.nl/gpt/.

[54] Borland, M. Elegant: A Flexible SDDS-Compliant Code for Accelerator Simulation, APS Tech Note LS-207 (2000).

[55] Borland M (2001). Simple method for particle tracking with coherent synchrotron radiation. Phys. Rev. Special Topics – Accel. Beams.

[56] Minty, M. G. & Zimmermann, F. Beam Techniques–Beam Control and Manipulation, Report No. SLAC-R-621 (2003).

[57] Casalbuoni, S., Schmidt, B. & Schmüser, P. Far-Infrared Transition and Diffraction Radiation, TESLA Report 2005–15 (2005).

[58] Hara T (2016). Pulse-by-pulse multi-beam-line operation for x-ray free-electron lasers. Phys. Rev. Accel. Beams.

[59] Faatz B (2016). Simultaneous operation of two soft x-ray free-electron lasers driven by one linear accelerator. New J. Phys..

[60] Emma P (2010). First lasing and operation of an angstrom-wavelength free-electron laser. Nature Photon..

[61] Ishikawa I (2012). A compact X-ray free-electron laser emitting in the sub-angstrom region. Nature Photon..

[62] Kang, H.-S. *et al*. Current Status of PAL-XFEL Project, In Proc. of the 4^th^ Intern. Part. Accel. Conf., 2074–2076 (2013).

[63] Altarelli M (2011). The European X-ray free-electron laser facility in Hamburg. Nucl. Instrum. Methods Phys Res. B.

[64] Milne CJ (2017). SwissFEL: The Swiss X-ray Free Electron Laser. Applied Sciences.

[65] Emma P (2014). Experimental Demonstration of Energy-Chirp Control in Relativistic Electron Bunches Using a Corrugated Pipe. Phys. Rev. Letters.

[66] Antipov S (2014). Experimental Demonstration of Energy-Chirp Compensation by a Tunable Dielectric-Based Structure. Phys. Rev. Letters.

[67] Deng H (2014). Experimental Demonstration of Longitudinal Beam Phase-Space Linearizer in a Free-Electron Laser Facility by Corrugated Structures. Phys. Rev. Letters.

[68] Antipov S (2012). Experimental Observation of Energy Modulation in Electron Beams Passing through Terahertz Dielectric Wakefield Structures. Phys. Rev. Letters.

