# Supplementary material for: Coherent THz Emission Enhanced by Coherent Synchrotron Radiation Wakefield
Source: Sci Rep. 2018 Aug 3;8:11661. doi: 10.1038/s41598-018-30125-1 (PMC6076281; doi:10.1038/s41598-018-30125-1)
Supplement: Supplementary file 1 — Supplementary Information [file 41598_2018_30125_MOESM1_ESM.pdf]

Coherent THz Emission Enhanced by Coherent Synchrotron Radiation Wakefield

S. Di Mitri[1,2], A. Perucchi[1], N. Adhlakha[1], P. Di Pietro[1], S. Nicastro[2], E. Roussel[1,3], S. Spampinati[1], M. Veronese[1], E. Allaria[1], L. Badano[1], I. Cudin[1], G. De Ninno[1], B. Diviacco[1], G. Gaio[1], D. Gauthier[1,4], L. Giannessi[1,5], S. Lupi[6,7], G. Penco[1], F. Piccirilli[7], P. Rebernik[1], C. Spezzani[1], M. Trovo'[1]

[1] *Elettra–Sincrotrone Trieste S.C.p.A., 34149 Basovizza, Trieste, Italy*
[2] *University of Trieste, Department of Physics, 34100 Trieste, Italy*
[3] *Univ. Lille, CNRS, UMR 8523 - PhLAM - Physique des Lasers Atomes et Molécules, 59000 Lille, France*
[4] *LIDYL, CEA, CNRS, Université Paris-Saclay, Saclay, 91191 Gif-sur-Yvette, France*
[5] *ENEA Frascati, Via E. Fermi 45, 00044 Frascati, Rome, Italy*
[6] *University of Rome – La Sapienza, 00185 P.le A. Moro 2, Rome, Italy*
[7] *CNR-IOM, 34149 Basovizza, Trieste, Italy*

I. ELECTRON BEAM DYNAMICS

This Section shows the evolution of the electron beam longitudinal phase space through the Main Beam Dump (MBD) line of the FERMI free-electron laser (FEL), as predicted by the `elegant` particle tracking code, in the presence of one-dimensional (1-D) coherent synchrotron radiation (CSR) emission. The signature of the CSR wakefield on the bunch longitudinal phase space for a flat-top current profile at the entrance of a dipole magnet is shown in Fig. S1. The simulated longitudinal phase space and current profile of the electron bunch corresponding to the experimental sessions are shown in Fig. S2 at the entrance, in the middle and at the end of the MBD line, respectively.

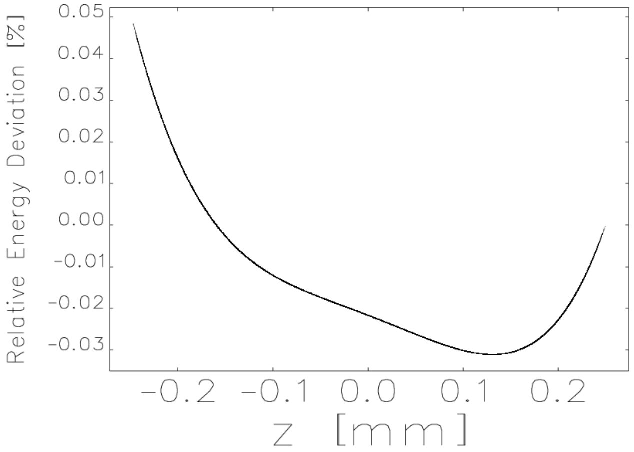

Supplementary Figure S1. Relative energy deviation along the electron bunch per meter induced by CSR wakefield in a dipole magnet. Bunch head is at negative z-coordinates. A 700-pC total bunch charge and hard-edge flat-top current profile at the beam mean energy of 1 GeV is assumed. The linear component of the energy chirp in the bunch head is $h \cong 7\ m^{-1}$, and a chirp approximately 3.5 times larger would be expected at the end of MBD line (the total arc length in the dipoles is 3.56 m). See also Section II of the main manuscript.

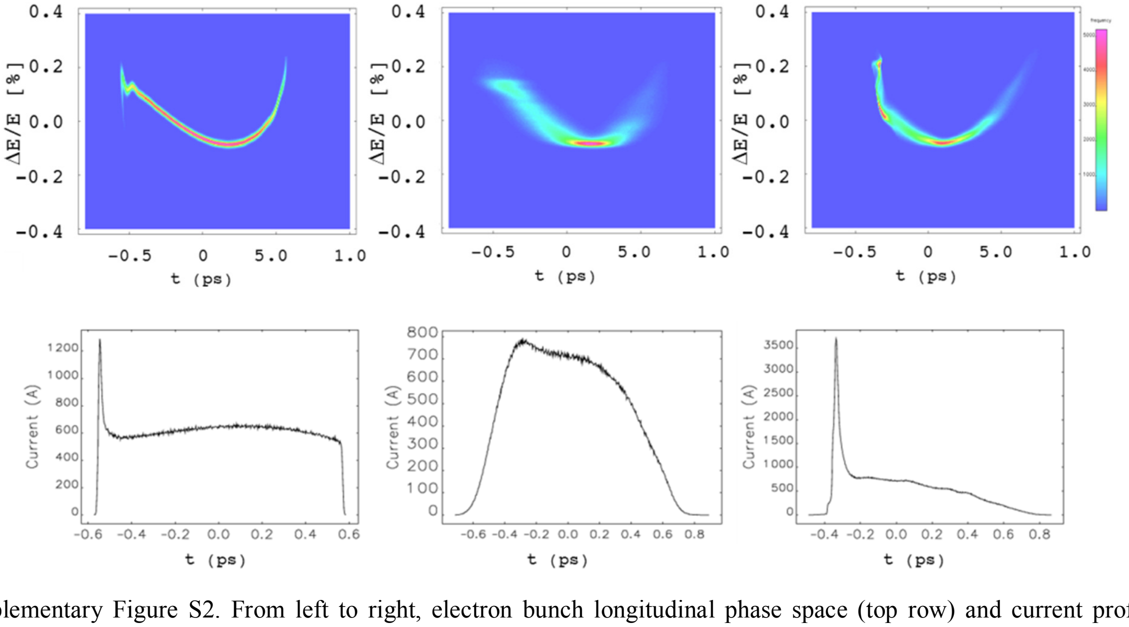

Supplementary Figure S2. From left to right, electron bunch longitudinal phase space (top row) and current profile (bottom row) at the entrance of the MBD line, after "Dipole 1", and after "Dipole 2" (see also Fig.1 in the main manuscript). Particle tracking results obtained with the `elegant` code, including 1-D modelling of CSR wakefield. Electron beam and linac parameters are in Tab.1 of the main manuscript, for the beam energy E=1308 MeV at the linac end.

II. CALCULATED THZ PULSE PROPERTIES

This Section reproduces Figs. 3 and 4 in the main manuscript, now with linear scale for the frequency and with bandwidth normalization of the calculated pulse energy. These plots provide additional quantitative information on the expected spectral brilliance of the THz pulse.

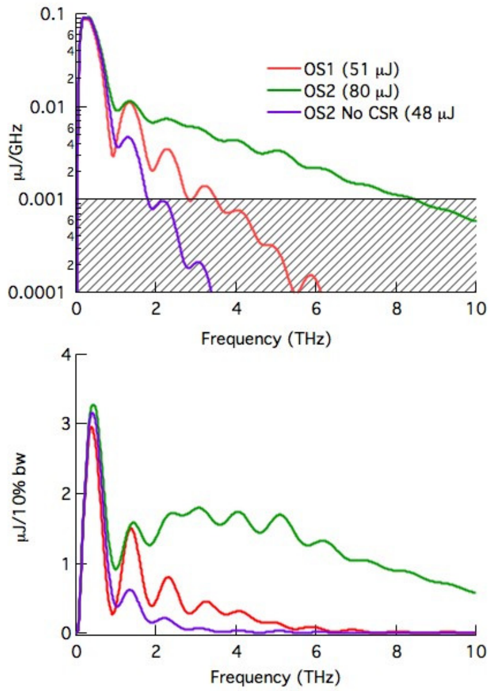

Supplementary Figure S3. Calculated pulse energy per GHz (top) and per 10% bandwidth (bottom), for two optics settings of the dump line (OS1-red and OS2-green). For comparison, a case without CSR in the dump line dipole magnets is shown (violet). See also Fig. 3 in the main manuscript.

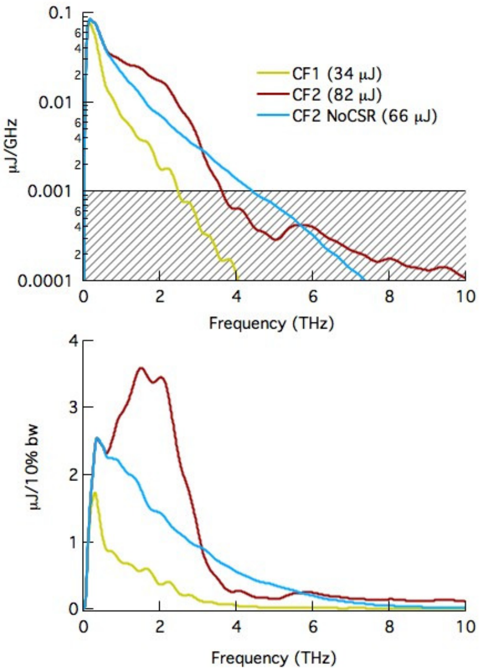

Supplementary Figure S4. Calculated pulse energy per GHz (top) and per 10% bandwidth (bottom), for two compression factors in the linac (CF1-yellow and CF2-brown) and fixed R_{56} in the dump line. For comparison, a case without CSR in the dump line dipole magnets is shown (cyan). See also Fig.4 in the main manuscript.

III. EXPERIMENTAL THZ SPECTRA

We report in this section two examples of spectra measured in the TeraFERMI laboratory with the help of a Fourier Transform Infrared (FTIR) step-scan spectrometer. The spectra were acquired at different settings of the FERMI beam delivery system, and demonstrate that multi-THz emission can indeed be generated at FERMI in parasitic mode to the FEL. These results were not measured in correspondence of the data shown in the main manuscript so that a direct comparison with the simulations does not hold.

It is also important to keep in mind that the spectral content is affected by the convolution of the THz pulse with the optical response of the various transport elements bringing the light to the FTIR spectrometer located in the experimental hall, 30 m downstream the THz source. The spectra were measured in air, so that water vapour absorption also contributes.

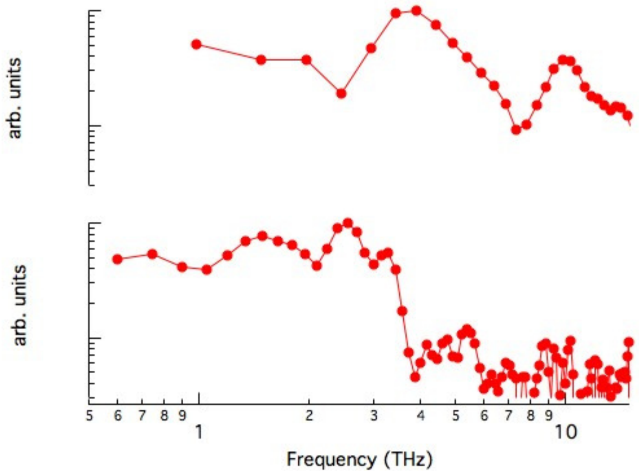

Supplementary Figure S5. Two examples of experimental FTIR spectra acquired with different machine settings at the TeraFERMI laboratory. FERMI settings during these measurements were not the same of those described in the manuscript, so that a direct comparison does not apply.
